# Characterization and Differentiation of *Candida auris* on Dixon’s Agar Using Raman Spectroscopy

**DOI:** 10.3390/pathogens13110978

**Published:** 2024-11-08

**Authors:** Chrysoula Petrokilidou, Eleftherios Pavlou, Aristea Velegraki, Anna Simou, Ioanna Marsellou, Grigorios Filis, Ioannis D. Bassukas, Georgios Gaitanis, Nikolaos Kourkoumelis

**Affiliations:** 1 Department of Medical Physics, Faculty of Medicine, University of Ioannina, 451 10 Ioannina, Greece; 2 Mycology Laboratory, BIOIATRIKI SA, 115 27 Athens, Greece; 3 Hautwerk Klinik, 8041 Zürich, Switzerland; 4 Department of Skin & Venereal Diseases, Faculty of Medicine, University of Ioannina, 451 10 Ioannina, Greece

**Keywords:** Raman spectroscopy, *candida*, *candida auris*, modified Dixon’s agar, spatially offset Raman spectroscopy, SORS

## Abstract

*Candida auris*, an emerging multidrug-resistant fungal pathogen, poses significant challenges in healthcare settings due to its high misidentification rate and resilience to treatments. Despite advancements in diagnostic tools, a gap remains in rapid, cost-effective identification methods that can differentiate *C. auris* from other *Candida* species, particularly on non-standard culture media. We used Raman spectroscopy to characterize *C. auris* grown on modified Dixon’s agar (mDixon) and differentiated it from *Candida albicans* and *Candida parapsilosis*. Key Raman spectral markers at 1171 cm^−1^ and 1452 cm^−1^, linked to mannan and β-glucan composition, differentiated *C. auris* into two subgroups, A and B. Despite the spectral similarities of groups A and B with *C. albicans* and *C. parapsilosis*, respectively, all *Candida* species were distinguishable through principal component analysis (PCA). Additionally, this study is the first to demonstrate the distinct spectral signature of mDixon agar, achieved through spatially offset Raman spectroscopy (SORS), which enables accurate discrimination between the culture medium and fungal samples. The observed inter-individual variability within *C. auris*, coupled with the spectral overlap between *C. auris* subgroups and other *Candida* species, highlights a major challenge in differentiating closely related fungi due to their similar molecular composition. Enhancements in spectral resolution and further fluorescence minimization from the culture medium are needed to reliably detect the subtle biochemical differences within these species. Despite these challenges, the results underscore the potential of Raman spectroscopy as a real-time, non-destructive, and complementary tool for fungal pathogen identification.

## 1. Introduction

*Candida auris* is an emerging fungal pathogen recently identified by the World Health Organization (WHO) as a critical priority group pathogen among *Cryptococcus neoformans*, *Candida albicans*, and *Aspergillus fumigatus* [1]. The United States Centers for Disease Control and Prevention (CDC) also listed *C. auris* an urgent and critical public health threat [2]. The pathogen is known for causing nosocomial outbreaks exhibiting increased resistance to antifungal drugs compared to other *Candida* species [3,4,5,6]. *C. auris* can colonize the skin surface with preference for specific anatomical sites like the nostrils, axillae, and inguinal folds [7]. In these skin regions, similar to the preferred environments of lipophilic yeasts from the genus *Malassezia*, *C. auris* finds conducive conditions for establishing persistent colonization and creating a reservoir of yeasts. This reservoir may serve as a source of pathogens that could lead to subsequent infection episodes in the affected patient [8,9]. The competitive interaction between these two yeast genera for colonization of specific niches in humans is evident from observations that in *C. auris*-negative individuals, the fungal microbiota is dominated by *Malassezia* species while *C. auris*-positive individuals have a fungal microbiota dominated by *Candida* species such as *C. auris* and *C. albicans*, among other microorganisms [10]. Although this finding could also suggest that the host microbiome may play a crucial role in preventing *C. auris* colonization, it is still unclear whether incidental dysbiosis precedes *C. auris* colonization or not. The pathogen’s robust nature and the challenges it poses in healthcare settings emphasize the need for accurate identification and effective management strategies. Under laboratory conditions *C. auris* typically grows on standard Sabouraud-Dextrose agar [11]. However, in routine chromogenic agars, it is often mistaken for other clinically significant *Candida* species [12], necessitating the use of more sophisticated and costly methods such as matrix-assisted laser desorption/ionization time-of-flight mass spectrometry (MALDI-TOF MS) in both research and clinical settings. Thus, a major challenge in addressing *C. auris* infections is its frequent misidentification in routine diagnostic assays, often due to similarities with other *Candida* species on standard culture media.

Raman spectroscopy has emerged as a powerful tool for the differentiation of pathogens, including *C. auris* [13,14,15,16,17,18,19,20] and *Malassezia* spp. [21]. By analyzing the vibrational modes of molecular components, it can distinguish between different species based on their unique spectral fingerprints. Raman spectroscopy presents several advantages over MALDI-TOF mass spectrometry, including non-destructive analysis, operational simplicity, speed, and minimal sample preparation. Unlike more complex techniques that require extensive sample preparation, Raman spectroscopy can analyze samples in situ, making it a practical complement to the commonly used diagnostic methods. However, it has not yet been widely implemented in clinical laboratories, largely due to challenges such as fluorescence interference from culture media and the need for standardized, yet optimized, analysis protocols across varying experimental setups. Conversely, the non-destructive nature of Raman spectroscopy is essential for preserving sample integrity, especially when the samples need to be available for further analysis. It also reveals the biochemical composition and metabolic state of the microorganisms, including lipids, proteins, and nucleic acids. Additionally, it offers functional insights into their physiological conditions, including stress response and the presence of specific metabolites. This contrasts with MALDI-TOF MS, which is superior in terms of sensitivity and specificity for organism identification. Supported by extensive microbial databases, it is highly effective for precise species and strain-level identification in routine clinical laboratories. On the other hand, Raman spectroscopy offers a rapid (within seconds) method for identifying and classifying yeasts like *C. auris*, aiding the effective management of infections caused by such pathogens.

In this study, we investigate whether Raman spectroscopy can distinguish *C. auris* from other *Candida* species, even on non-standard culture media like modified Dixon’s agar (mDixon), offering a potentially fast and cost-effective diagnostic alternative. Specifically, we focus on (i) the Raman characterization of *C. auris* on mDixon agar and (ii) its comparison to *C. parapsilosis*, a WHO-designated high-priority pathogen, and *C. albicans*, both of which, like *C. auris*, frequently colonize skin folds. The goal is to develop a rapid, non-contact method to accurately identify and differentiate each pathogen on a non-standard culture medium, with the potential for implementation in routine clinical diagnostics.

## 2. Materials and Methods

### 2.1. Isolation and Cultivation of Candida Species

Thirty-three *Candida* cultures, including different clinical isolates of *C. auris* (n = 20), *C. parapsilosis* (n = 7), and *C. albicans* (n = 6). Three samples of mDixon were also measured. *C. auris* strains were sourced from humans from diverse clinical material: blood from central lines and areas surrounding surgically treated abdominal aortic aneurysms. Specimens related to catheter use were taken from venous catheters and jugular lines. Bronchial and respiratory secretions were obtained to investigate respiratory tract infections. Specimens were also collected from wound and surgical sites from urinary tract infections and from axillary and groin regions, including catheter sites. The majority of *C. parapsilosis* and *C. albicans* strains originated from nail specimens while one of them was from the coccyx region. All strains were cultured on modified Dixon’s (mDixon) agar [22] for 14 days at a temperature of 32 °C. *C. auris* strains were characterized by MALDI-TOF MS analysis at log scores 1.9–2. It is well-documented that in our geographic region, *C. auris* Clade I is the most prevalent [23]. Based on the geographic origin and clinical context, it is reasonable to suggest that the Raman spectroscopy data we collected likely differentiate between strains of *C. auris* Clade I, as this clade is the most prevalent in the region. However, confirming whether the Raman profiles specifically correlate with Clade I strains requires further investigation, including genetic validation to verify clade identity alongside Raman spectroscopy. Genetic confirmation was beyond the scope of this study, which focused primarily on spectral characterization. Future studies could integrate genetic analysis to provide a more comprehensive understanding of *C. auris* variability. The *C. auris* population studied here exhibited differences in their protein profiles, suggesting that while the genetic material is the same, the protein expression or post-translational modifications vary among samples, potentially due to environmental factors or other influences affecting protein synthesis. Therefore, in this study we will concentrate on the Raman characterization of the structural biopolymers of the fungal cell wall, namely the glucans, chitin, and mannans. Although we cannot entirely rule out contributions from lipid signatures in the Raman spectra originating from the cell membrane, *Candida* species exhibit similar levels of various phospholipid classes [24]. However, each species presents a distinct molecular lipid species profile, which is reflected in their unique mass spectrometry imprints but is not expected to be manifested in the heights of the Raman bands.

### 2.2. Raman Spectroscopy Setup

Raman spectroscopy is a technique that identifies molecular structures by analyzing the scattering of laser light after interacting with the chemical bonds within a sample, revealing unique vibrational patterns. This spectral “fingerprint”, enables differentiation among samples based on their molecular composition. The Raman setup consisted of a laser (Mini-Benchtop Stabilized Laser, Coherent, Santa Clara, CA, USA) operating at 785 nm with a maximum power output of 300 mW, and an f/1.3 Raman spectrometer (WP 785 ER Raman Spectrometer, Wasatch Photonics) with the excitation and collection of optical fibers coupled to a single probe tip (Wasatch Photonics, Morrisville, NC, USA). The Raman spectra were collected directly from multiple regions of substantial yeast growth on the culture plate, without any processing prior to measurement. Each spectrum was the result of averaging 5 spectra from the same point, each with an acquisition time of 5 s, while the laser power was properly adjusted to avoid saturation of the spectrometer’s charge-coupled device (CCD). A dark noise spectrum was recorded before each measurement and was automatically subtracted by the recording software (Enlighten Spectroscopy software v 2.5.7, Wasatch Photonics, USA).

Additionally, an in-house spatially offset Raman spectroscopy (SORS) setup [25,26] was configured to measure uncultured mDixon agar, which was used as the reference. In this case, SORS was used as an intensity reduction technique to mitigate the high levels of CCD saturation due to the fluorescence caused by the agar’s constituents even at the lowest laser power setting. Fluorescence, typically more intense at the surface or near-surface layers of a material, often overwhelms the weaker Raman signals, hindering the ability to accurately detect and analyze the sample’s chemical composition. SORS effectively bypasses this problem by spatially separating the Raman signal collection point from the laser excitation point. The spatial offset allows the technique to probe deeper layers within the sample, where fluorescence may be less prominent, thereby reducing its interference with the Raman signal [27]. Moreover, the inherent spectral differentiation achieved through SORS enables the extraction of more intense Raman signals by mitigating the fluorescence background, making it particularly useful for samples with high intrinsic fluorescence like Dixon’s agar. The SORS setup was the same as the setup used for measuring the yeasts, with the only difference being the use of a separate excitation probe (BAC102, B&W TEK Newark, DE, USA) at a 45° angle with respect to the sample, producing a defocused beam of elliptical shape (with major and minor axes of approximately 3 mm and 2 mm, respectively) on the sample. We collected three spectra from the agar, directly from the culture plate, at spatial offset of 2 mm, with the same conditions as in the yeasts’ case (acquisition of 5 s, averaging of 5 spectra, with dark noise subtraction).

### 2.3. Data Acquisition and Analysis

Before analysis, all spectra were cropped to the fingerprint region (400–1800 cm^−1^) and were subject to spike removal, Savitzky-Golay smoothing using a 2nd degree polynomial with a 17 points window, baseline removal using the SNIP algorithm [28], and unit vector normalization. These preprocessing steps were applied to enhance data quality by minimizing noise and baseline variation, while preserving the most relevant spectral features. This ensures the data is suitable for dimensionality reduction and clustering. All procedures, along with principal components analysis (PCA), were performed using in-house developed software [29]. PCA is a statistical technique used to simplify complex datasets by reducing their dimensions while retaining the most significant information. In PCA, new variables called principal components (PCs) are created. Each PC captures a different percentage of variability within the data, with the first PC representing the direction of greatest variance, followed by subsequent PCs in decreasing order of variance. A PCA scores plot visualizes how individual samples or groups of data are positioned in the space defined by the PCs, allowing for patterns or clusters to be observed, which can differentiate species or sample types. The loadings plot, on the other hand, shows how each original variable (such as specific Raman spectral bands) contributes to the PCs, highlighting the key features responsible for the observed separation in the scores plot. Together, these tools make it possible to identify and interpret the main factors that distinguish different groups in complex datasets, such as the biochemical variations among *Candida* species. PCA was implemented using the scikit-learn library in Python. The first two principal components, which captured the most variability in the dataset, were plotted for visualization, with clusters corresponding to different Candida groups. The separation between groups was further confirmed by applying linear discriminant analysis (LDA) on the PCA-transformed data. The LDA decision boundaries maximize the separation between clusters providing both visual and quantitative validation of the *Candida* species discrimination. To further validate species-level differences in spectral features, we applied Welch’s *t*-test to key Raman band intensity ratios. This test was chosen for its robustness in handling groups with unequal variances and sample sizes, rendering it suitable for comparisons between the *Candida* species studied in this work.

## 3. Results

**Figure 1:**
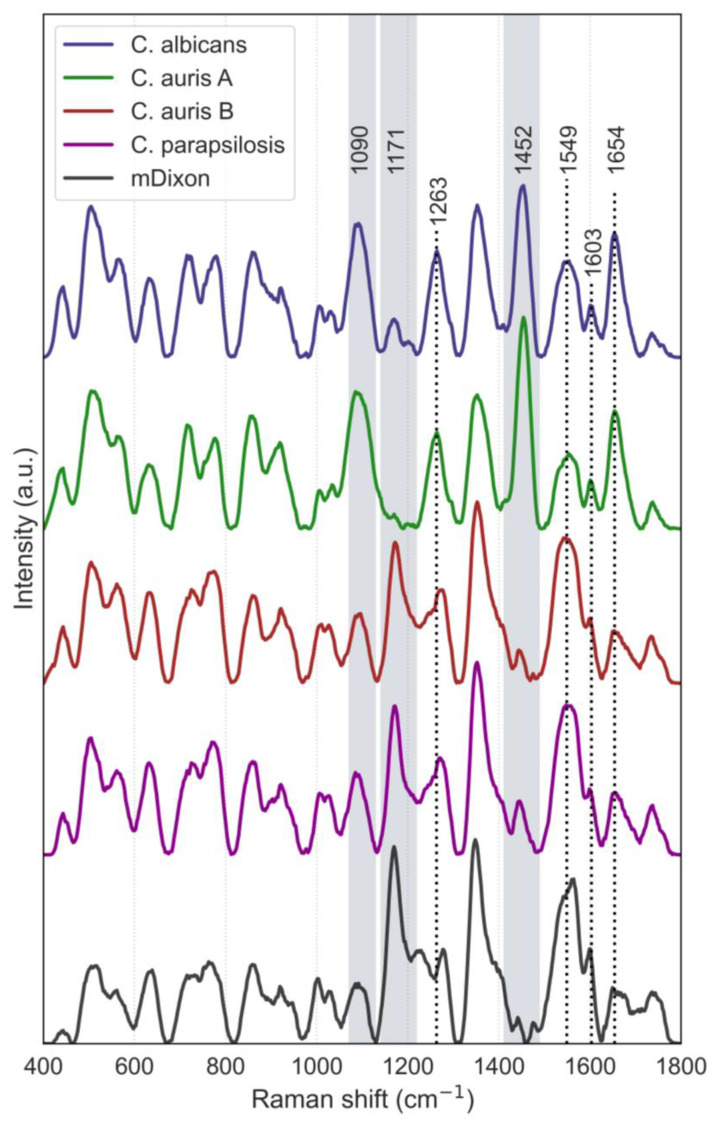
Average Raman spectra of the three *Candida* species and the culture medium (mDixon). The shaded regions in the spectrum highlight the three Raman bands that are key to distinguishing the characteristic differences between the species. Dotted lines mark other vibrational modes associated with the molecular components of the cell walls of the *Candida* species.

Spectral preprocessing of the *C. auris* Raman spectra revealed significant inter-individual peak variation suggesting the allocation of the specimens into two different groups: one with Raman spectra similar to those of *C. albicans* and the other resembling those of *C. parapsilosis*. This subdivision, designated as *C. auris* A and *C. auris* B, was further supported by the inverted intensities of the Raman bands at 1171 and 1452 cm^−1^ (Figure 1), which represent vibrational modes associated with specific biochemical components of *Candida* species. These components are meaningful because they vary between species, providing unique spectral “fingerprints” that can aid in identifying fungal pathogens. Subgroup A consisted of 5 cultures, while subgroup B comprised 15 cultures. Moreover, this separation was evident macroscopically: dense colonies formed in *C. auris* A, whereas *C. auris* B exhibited more discrete, well-developed colonies. The average Raman spectra from culture probes of the three *Candida* species, including the two spectral variations of *C. auris* and the growth medium (mDixon), are displayed in Figure 1.

The Raman spectra presented in Figure 1 are the spectral fingerprints of *C. albicans*, *C. auris* (groups A and B), *C. parapsilosis*, and mDixon agar medium. The marked bands at approximately 1090 cm^−1^, 1171 cm^−1^, 1263 cm^−1^, 1452 cm^−1^, 1549 cm^−1^, 1603 cm^−1^, and 1654 cm^−1^ correspond to key vibrational modes associated with the molecular components of the cell walls of the *Candida* species. The band at 1090 cm^−1^ is typically attributed to C-O stretching vibrations in β-glucans, suggesting a strong presence of this polysaccharide in the cell walls across the different species. The 1171 cm^−1^ band, prominent in *C. parapsilosis* and *C. auris* B, is linked to C-H deformation and C-O stretching vibrations, indicative of mannans. The 1263 cm^−1^ band, visible across all *Candida* spectra, can be associated with amide III vibrations, reflecting the presence of chitin, which is critical for structural integrity, particularly in the hyphal forms associated with invasive growth. The 1452 cm^−1^ band, evident in *C. albicans* and *C. auris* A, is related to CH_2_ bending vibrations, often attributed to both polysaccharides and lipids, indicating differences in cell wall composition and possibly reflecting structural adaptations. The bands at 1549 cm^−1^ and 1654 cm^−1^ correspond to amide II and amide I vibrations, respectively, both of which are related to the presence of chitin and other protein components in the cell wall. The 1603 cm^−1^ band, though less common in polysaccharides, could indicate aromatic ring stretching potentially related to phenolic compounds or interactions with aromatic residues in proteins. Based on *Candida*’s cell wall composition, a tentative assignment of the major bands is shown in Table 1.

**Figure 2:**
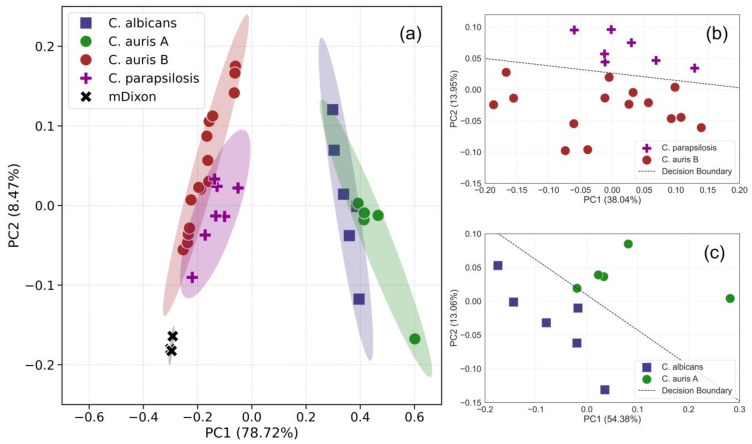
(**a**): PCA scores plot of the first two principal components (PC1 and PC2). The shaded regions represent the 95% confidence ellipses of the respective classes; (**b,c**): Additional PCA plots focusing on pairs of closely related clusters: (**b**) *C. auris* B vs. *C. parapsilosis*, and (**c**) *C. albicans* vs. *C. auris* A. In both cases, a linear discriminant analysis (LDA) decision boundary was calculated using the scores from the first two principal components. The decision boundary highlights the separation achieved by LDA between the groups based on their PC scores, further supporting the distinction between the *Candida* species.

**Figure 3:**
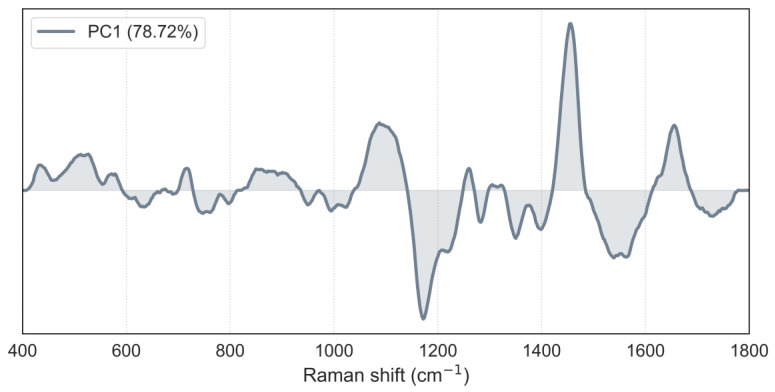
PC1 loadings plot representing 78.72% of the variance in the Raman spectral data. The loadings plot indicates the contribution of each Raman shift to PC1, with positive and negative loadings highlighting the key spectral regions responsible for the separation of the samples in the PCA analysis.

As previously noted, *C. auris* spectra reveal two distinct groups with different Raman bands heights: one with Raman spectra similar to *C. albicans* and the other resembling *C. parapsilosis*, leading to potential misidentification between these species. However, PCA analysis (Figure 2) demonstrates that all *Candida* classes (and mDixon) can be distinctly clustered by the first two principal components. However, the presence of multiple clusters from different *Candida* species and mDixon in Figure 2a can obscure the finer discrimination of the *C. auris* subgroups due to visual overlap and the dataset’s density. This is a common limitation in global PCA visualizations when projecting multiple groups into the same low-dimensional space. To better resolve these differences, we conducted pairwise PCA analyses (Figure 2b,c), focusing on closely related groups. These pairwise plots demonstrate clear separations, with distinct clustering between *Candida* groups driven by the unique features of the Raman spectra. Additionally, the use of LDA decision boundaries quantitatively demonstrates this separation, offering both a visual and statistical validation of the subgroup discrimination. PC1 accounts for ~78% of the variance, and the loadings plot (Figure 3) indicates that the bands at 1090, 1171, and 1452 cm^−1^ are primarily responsible for this differentiation.

**Figure 4:**
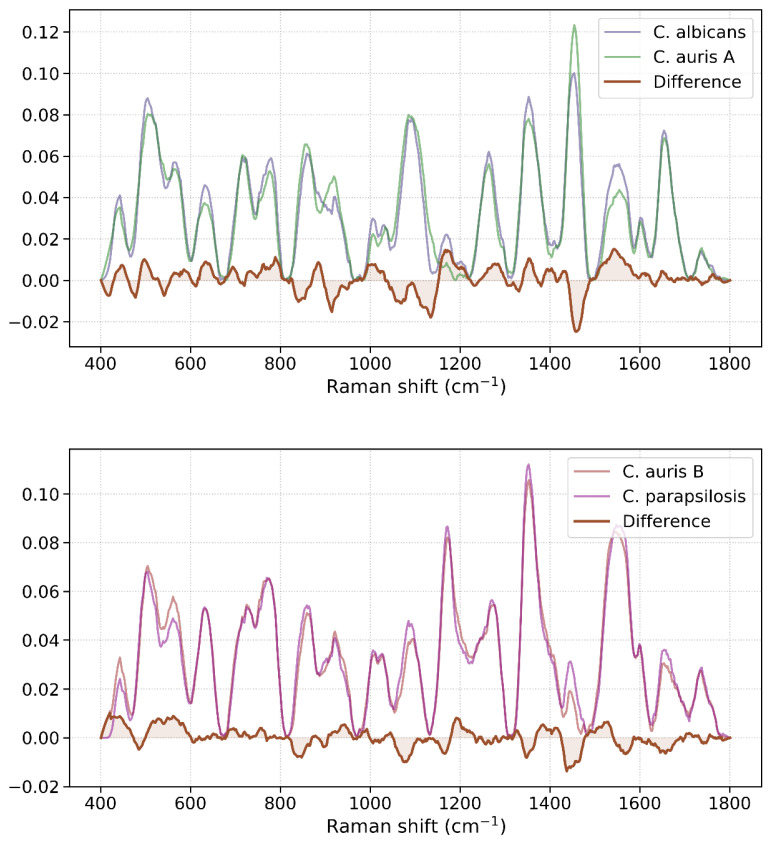
Difference spectra of *C. albicans* vs. *C. auris* A and of *C. auris* B vs. *C. parapsilosis*.

This finding is further corroborated by the difference spectra of *C. auris* A vs. *C. albicans* and *C. auris* B vs. *C. parapsilosis* (Figure 4). In Figure 4, the Raman shifts (positions of the peaks) appear to be largely consistent between the species within each pair, indicating that the basic molecular structures producing these vibrational modes are similar. However, the difference spectra reveal variations in the amplitude or intensity of these bands, particularly around the ~1452 cm^−1^ region, as well as in other areas to a lesser extent. This suggests differences in the concentration or environment of the molecular species responsible for these vibrational modes. Although the Raman signal contour of the spectra is similar due to shared structural components like β-glucans and mannans, the differences in the 1452/1090 cm^−1^ (CH_2_ bending vibrations to β-glucans) and 1452/1171 cm^−1^ (CH_2_ bending vibrations to mannans) ratios are key to distinguishing the species. By analyzing the ratios of these bands, we can infer differences in the yeasts’ cell wall composition.

Furthermore, it is important to note that mDixon medium can be distinctly identified within its own cluster (Figure 2), serving as a control and ensuring that the Raman beam does not penetrate the sample to include spectral features of the medium. To our knowledge, this is the first time this medium (i) has been used as a control for *Candida* species and (ii) assigned to a Raman spectrum.

## 4. Discussion

This study demonstrates the potential of the Raman spectroscopy as a feasible approach to the identification of *Candida* strains grown on mDixon and to their characterization at species level based on the recognition of variations in characteristic spectral band features. These latter decisive bands are likely associated with (exo-)polysaccharides, which are major components of the cell wall in *Candida* species [15]. The cell wall’s primary components (β-glucans, mannans, and chitin) vary in concentration, structure, and conformation among these species, leading to variations in the Raman bands’ intensities. The primary structural component of glucans is β-1,3-glucan, linked to β-1,6-glucan, which forms the scaffold for the cell wall. Glucans are synthesized by glucan synthases, which are essential for cell wall integrity and morphology [30]. Chitin is located in the inner layer providing mechanical strength. The chitin content increases in the hyphal form, which is crucial for invasive growth and infection [31]. Finally, the outer layer of *Candida* species consists of mannoproteins with N-linked and O-linked mannans, which play a significant role in immune evasion by masking the underlying β-glucan, thereby preventing detection by the host immune system. This masking effect is crucial for the pathogenicity of *Candida*, as it helps the organism evade immune recognition and response. N-linked mannans are particularly effective in concealing β-glucan from recognition by Dectin-1, a pattern recognition receptor on host immune cells. Additionally, the absence or modification of these mannans results in increased immune detection and phagocytosis of *Candida* cells, highlighting their protective role [32]. These compositional differences shape each species’ cell wall to enhance survival, pathogenicity, and resistance to environmental stress [33].

The two distinct clusters observed in the PCA plot of *C. auris* grown on a single mDixon medium are indicative of significant phenotypic plasticity within the species, likely driven by environmental factors and experimental conditions. Specifically for *C. auris*, it has been previously observed that after 24–48 h incubation, confluent growth is noted at heavy inoculum plating on the agar surface, with individual colonies not easily distinguishable, while single colonies appear on plates at lower inoculum size [34]. This pattern was also observed in our case, where *C. auris* A exhibited the former behavior and *C. auris* B exhibited the latter, further elucidating the dual-class outcome in the PCA analysis of *C. auris*. At low inoculum sizes, growth of single colonies likely provides a more favorable environment for individual phenotypic development due to decreased competition and ample resource availability. Conversely, high inoculum sizes promote dense confluent growth, where the cells are subjected to intense competition for nutrients and space, leading to more uniform stress responses and metabolic adaptations across the population. Small microenvironmental variations across the agar plate, such as slight fluctuations in nutrient concentration, pH, or moisture levels, could have induced these variations. Phenotypic switching in *Candida* species is also a well-documented adaptive mechanism where the organism can alter its physical appearance and/or functional attributes in response to environmentally triggered phenomena. Furthermore, *C. auris* was shown to be capable of altering its colony morphology when cultured on CHROMagar *Candida* [35]. That study found that the frequency of phenotypic switching varies among different *C. auris* strains, and the switching process was reversible, with certain phenotypes exhibiting greater stability once formed. In this context, the observed differences and classifications in the PCA plot seem to be driven by phenotypic variations, which still require further investigation. However, this finding implies that the metabolic and phenotypic variability observed among the samples is attributable solely to environmental influences and experimental variables, emphasizing the impact of non-genetic factors on *Candida* behavior. Additionally, differences in growth rate among the colonies could also contribute to the observed phenotypic differences as the cultures were not synchronous. The growth rate of fungal colonies can significantly affect their metabolic profile and the accumulation of secondary metabolites, which are detectable by Raman spectroscopy. Colonies that grow faster may exhaust nutrients more quickly and accumulate metabolic byproducts at different rates compared to slower-growing colonies. This could lead to alterations in cellular composition and function that manifest in the Raman spectral data, contributing to the separation seen in PCA. In the PCA plot, the proximity of the *C. auris* classes to *C. parapsilosis* and *C. albicans*, despite their clear separation from each other, suggests subtle metabolic similarities between these species that may not be immediately apparent from their genetic profiles. While the Raman spectra of *C. auris*, *C. albicans*, and *C. parapsilosis* may appear similar, as expected given their shared biochemical profile, it is the differences in specific Raman band ratios that allow us to distinguish between these species. For *C. auris* B vs. *C. parapsilosis*, the 1452/1090 cm^−1^ ratio, which highlights differences in β-glucan composition, indicates either a modification in the structural arrangement or a diverse concentration of lipids relative to β-glucans. For *C. auris* A vs. *C. albicans*, the 1452/1171 cm^−1^ ratio, related to mannan structure, indicates variations in mannan branching or the glycosidic linkages potentially altering the surface properties of the cell. Statistical analysis using Welch’s *t*-test demonstrated that these ratios are significantly different (*p* < 0.05) (Supplementary Materials), confirming the effectiveness of Raman spectroscopy for species-level differentiation. These subtle but statistically significant differences reflect important biochemical variations in the relative abundance of polysaccharides like β-glucans and mannans, which play a critical role in maintaining the structural integrity of the *Candida* species’ cell wall.

*C. auris*, represented by two subgroups, A and B, shows distinct but related spectral features, suggesting certain variability in cell wall composition within this species. This variability could be due to differences in the proportion and structure of cell wall polysaccharides like β-glucans, mannans, and chitin. Our findings show considerable variability in the Raman spectra of *C. auris*, which we attribute primarily to phenotypic plasticity rather than experimental variability. Given that phenotypic plasticity is well-recognized in the literature as a major factor influencing molecular differences among *C. auris* samples [34], and considering that all samples were prepared and measured using consistent protocols, we believe this biological variation is the key factor driving the observed spectral differences. Although minor environmental fluctuations during culturing may have impacted phenotypic expression to some degree, the distinct spectral markers we identified are consistent with documented biochemical differences that distinguish *C. auris* from other Candida species, particularly in cell wall composition. To that end, Raman spectral fingerprints reveal that *C. albicans* has a higher β-glucan content relative to mannans, as indicated by the more intense band at 1452 cm^−1^. In contrast, *C. parapsilosis* exhibits a stronger band at 1171 cm^−1^, suggesting different mannose branching patterns or types of glycosidic linkages. Vibrational band intensity differences, as shown by plotting the difference spectra of the species with similar Raman contours (Figure 4), are indicative of variations in the abundance of specific molecular components rather than differences in the types of bonds or functional groups present. These variations could also reflect differences in the metabolic activity, growth conditions, or cellular composition between the species, which are crucial for distinguishing such species, as evident in the PCA score plot (Figure 2) and supported by the literature on *Candida* species [11,12]. A distinctive characteristic of the *C. auris* cell wall was suggested to be its unique mannan structure, marked by a mannose-α-1-phosphate side chain [36]. However, this unique structural feature is not detectable using a conventional Raman setup, particularly in the absence of appropriate standards.

In this study, we have also successfully conducted the first Raman measurement of modified Dixon’s agar, given the substrate’s tendency to produce significant fluorescence, complicating spectral measurements when used as control. By employing the SORS Raman technique to reduce fluorescence interference, we have captured the spectral signature of Dixon’s agar, as evidenced in both the PCA plot and the specific spectral bands observed. Notably, the band at 1220 cm^−1^ in the Raman spectrum of mDixon is not overlapped by any *Candida* species spectrum. This peak is likely attributed to C-N stretching vibrations commonly associated with proteins and peptides, components often found in agar compositions used for culturing yeasts. This identification confirms the successful isolation of the agar’s intrinsic spectral characteristics from the fungal samples, ensuring that the observed *Candida* spectra are not confounded by the substrate.

mDixon primarily supports the growth of lipid-dependent fungi like *Malassezia* species by providing essential lipid sources such as oleic acid and tween 40 or 80. In contrast, *Candida* species are not typically lipid-dependent, relying more on carbohydrate sources for their energy needs and cellular functions, though they can grow on mDixon agar. While *Candida* species do not primarily metabolize lipids for growth, the availability of lipids in the medium could potentially enhance the structural integrity of their cell membranes. Lipids, particularly sterols like ergosterol, are crucial components of fungal cell membranes, contributing to membrane fluidity and integrity [37]. Although *Candida* species do not rely on external lipid sources for the biosynthesis of ergosterol, the presence of lipids in the environment might still facilitate certain aspects of membrane assembly or modification under stress conditions. This could lead to altered growth patterns or cellular responses compared to growth on non-lipid-containing media, potentially enhancing the classification distance among the different *Candida* species [38].

## 5. Conclusions

In this study, we used Raman spectroscopy to examine the biochemical composition of Candida species grown on modified Dixon’s agar. By analyzing the unique spectral “fingerprints” produced by each species, we identified differences in cell wall components, allowing for species-level differentiation. The Raman spectral analysis highlights the biochemical diversity within the cell walls of various *Candida* species, crucial for their survival, pathogenicity, and resilience against environmental stress. Furthermore, this analysis underscores the difficulties in distinguishing between closely related *Candida* species, such as the different classes of *C. auris* that exhibit overlapping spectral features. Nevertheless, the structural complexity of the polysaccharides in the cell walls—such as branching degrees, types of glycosidic linkages, and polymerization levels—contribute to the unique Raman profiles observed. Notably, this study is the first instance of *C. auris* being cultured in modified Dixon’s media, which is a lipid-based medium. The Raman spectra profiles for *C. albicans* and *C. parapsilosis* as well as that of the medium were provided, demonstrating Raman spectroscopy’s capacity to distinguish between the substrate and each characteristic fungal spectral profile. While this study demonstrates the capability of Raman spectroscopy to detect subtle but meaningful differences in the cell wall composition of *Candida* species, its full potential as a reliable diagnostic tool in clinical settings requires further optimization and validation across larger sample sizes and additional species. However, our findings suggest that Raman spectroscopy is a promising and rapid complementary technique, offering real-time and non-destructive analysis that can potentially be performed prior to established diagnostic approaches.

## Figures and Tables

**Table 1:** Tentative assignment of the prominent Raman bands in *Candida* spectra and mDixon.

Raman Shift (cm^−1^)					Band Assignment
*C. albicans*	*C. auris* A	*C. auris* B	*C. parapsilosis*	mDixon	
1090	1090	1090	1090	1090	β-Glucans (C-O stretching)
1171	1171	1173	1171	1171	Mannans (C-O-H bending and C-H deformation)
-	-	-	-	1220	C-N stretching
1263	1263	1273	1270	1275	Chitin (Amide III, N-H bending)
1452	1454	1452	1450	1450	CH_2_ bending vibrations in polysaccharides (Mannans and β-Glucans) and lipids
1549	1553	1546	1555	1557	Chitin (Amide II, N-H bending)
1603	1601	1600	1600	1603	Aromatic C=C stretching vibrations
1654	1655	1655	1657	1653	Chitin (Amide I, C=O stretching)

## Data Availability

Data are available on request from the authors.

## Supplementary Materials

The following supporting information can be downloaded at: https://www.mdpi.com/article/10.3390/pathogens13110978/s1, This supplementary file contains the statistical analysis of the significant Raman band ratios for all *Candida* species included in the study. These ratios, which contribute to species differentiation in the relevant PCA results, provide a molecular basis for distinguishing between the different *Candida* species [39,40,41].

## References

[1] WHO (2022). Fungal Priority Pathogens List to Guide Research, Development and Public Health Action.

[2] Lyman M. (2021). *Notes from the Field*: Transmission of Pan-Resistant and Echinocandin-Resistant *Candida auris* in Health Care facilities―Texas and the District of Columbia, January–April 2021. MMWR Morb. Mortal. Wkly. Rep..

[3] Chowdhary A., Jain K., Chauhan N. (2023). *Candida auris* Genetics and Emergence. Annu. Rev. Microbiol..

[4] Geremia N., Brugnaro P., Solinas M., Scarparo C., Panese S. (2023). *Candida auris* as an Emergent Public Health Problem: A Current Update on European Outbreaks and Cases. Healthcare.

[5] Banik S. (2023). Editorial: *Candida auris*—Understanding the New Superbug. Front. Cell. Infect. Microbiol..

[6] Du H., Bing J., Hu T., Ennis C.L., Nobile C.J., Huang G. (2020). *Candida auris*: Epidemiology, Biology, Antifungal Resistance, and Virulence. PLoS Pathog..

[7] Montoya A.M. (2024). The Importance of *Candida auris* in Skin. Curr. Fungal Infect. Rep..

[8] Gaitanis G., Magiatis P., Hantschke M., Bassukas I.D., Velegraki A. (2012). The Malassezia Genus in Skin and Systemic Diseases. Clin. Microbiol. Rev..

[9] Velegraki A., Cafarchia C., Gaitanis G., Iatta R., Boekhout T. (2015). Malassezia Infections in Humans and Animals: Pathophysiology, Detection, and Treatment. PLoS Pathog..

[10] Tharp B., Zheng R., Bryak G., Litvintseva A.P., Hayden M.K., Chowdhary A., Thangamani S. (2023). Role of Microbiota in the Skin Colonization of *Candida auris*. mSphere.

[11] Franco L.C., Ahmed M., Kendra C.G., Sperling R.M., Van Benten K., Lavik J.-P., Emery C.L., Relich R.F., Gavina K. (2024). Validation of a Qualitative Real-Time PCR Assay for the Detection of *Candida auris* in Hospital Inpatient Screening. J. Clin. Microbiol..

[12] Preda M., Chivu R.D., Ditu L.M., Popescu O., Manolescu L.S.C. (2024). Pathogenesis, Prophylaxis, and Treatment of *Candida auris*. Biomedicines.

[13] Udupa R., Peralam Yegneswaran P., Lukose J., Chidangil S. (2024). Utilization of Raman Spectroscopy for Identification and Characterization of Fungal Pathogens. Fungal Biol. Rev..

[14] Ibelings M.S., Maquelin K., Endtz H.P., Bruining H.A., Puppels G.J. (2005). Rapid Identification of *Candida* spp. in Peritonitis Patients by Raman Spectroscopy. Clin. Microbiol. Infect..

[15] Maquelin K., Choo-Smith L.-P., Endtz H.P., Bruining H.A., Puppels G.J. (2002). Rapid Identification of *Candida* Species by Confocal Raman Microspectroscopy. J. Clin. Microbiol..

[16] Pezzotti G., Kobara M., Nakaya T., Imamura H., Asai T., Miyamoto N., Adachi T., Yamamoto T., Kanamura N., Ohgitani E. (2022). Raman Study of Pathogenic *Candida auris*: Imaging Metabolic Machineries in Reaction to Antifungal Drugs. Front. Microbiol..

[17] Pezzotti G., Kobara M., Nakaya T., Imamura H., Miyamoto N., Adachi T., Yamamoto T., Kanamura N., Ohgitani E., Marin E. (2022). Raman Spectroscopy of Oral *Candida* Species: Molecular-Scale Analyses, Chemometrics, and Barcode Identification. Int. J. Mol. Sci..

[18] Pezzotti G., Kobara M., Nakaya T., Imamura H., Fujii T., Miyamoto N., Adachi T., Yamamoto T., Kanamura N., Ohgitani E. (2022). Raman Metabolomics of *Candida auris* Clades: Profiling and Barcode Identification. Int. J. Mol. Sci..

[19] Pezzotti G., Kobara M., Asai T., Nakaya T., Miyamoto N., Adachi T., Yamamoto T., Kanamura N., Ohgitani E., Marin E. (2021). Raman Imaging of Pathogenic *Candida auris*: Visualization of Structural Characteristics and Machine-Learning Identification. Front. Microbiol..

[20] Fernández-Manteca M.G., Ocampo-Sosa A.A., Ruiz De Alegría-Puig C., Pía Roiz M., Rodríguez-Grande J., Madrazo F., Calvo J., Rodríguez-Cobo L., López-Higuera J.M., Fariñas M.C. (2023). Automatic Classification of *Candida* Species Using Raman Spectroscopy and Machine Learning. Spectrochim. Acta Part A Mol. Biomol. Spectrosc..

[21] Petrokilidou C., Pavlou E., Gaitanis G., Bassukas I.D., Saridomichelakis M.N., Velegraki A., Kourkoumelis N. (2019). The Lipid Profile of Three Malassezia Species Assessed by Raman Spectroscopy and Discriminant Analysis. Mol. Cell. Probes.

[22] Chermette R., Gueho E. (1994). Prévalence Du Genre Malassezia Chez Les Mammifères. J. Mycol. Médicale.

[23] Poulopoulou A., Sidiropoulou A., Sarmourli T., Zachrou E., Michailidou C., Zarras C., Vagdatli E., Massa E., Mouloudi E., Pyrpasopoulou A. (2024). *Candida auris*: Outbreak, Surveillance and Epidemiological Monitoring in Northern Greece. Med. Mycol..

[24] Singh A., Prasad T., Kapoor K., Mandal A., Roth M., Welti R., Prasad R. (2010). Phospholipidome of *Candida*: Each Species of *Candida* Has Distinctive Phospholipid Molecular Species. OMICS A J. Integr. Biol..

[25] Vardaki M.Z., Seretis K., Gaitanis G., Bassukas I.D., Kourkoumelis N. (2021). Assessment of Skin Deep Layer Biochemical Profile Using Spatially Offset Raman Spectroscopy. Appl. Sci..

[26] Vardaki M.Z., Pavlou E., Simantiris N., Lampri E., Seretis K., Kourkoumelis N. (2023). Towards Non-Invasive Monitoring of Non-Melanoma Skin Cancer Using Spatially Offset Raman Spectroscopy. Analyst.

[27] Conti C., Botteon A., Colombo C., Realini M., Matousek P. (2016). Fluorescence Suppression Using Micro-Scale Spatially Offset Raman Spectroscopy. Analyst.

[28] Morháč M., Kliman J., Matoušek V., Veselský M., Turzo I. (1997). Background Elimination Methods for Multidimensional Coincidence γ-Ray Spectra. Nucl. Instrum. Methods Phys. Res. Sect. A Accel. Spectrometers Detect. Assoc. Equip..

[29] Pavlou E., Kourkoumelis N. (2023). Preprocessing and Analyzing Raman Spectra Using Python. Eng. Proc..

[30] Estrada-Mata E., Navarro-Arias M.J., Pérez-García L.A., Mellado-Mojica E., López M.G., Csonka K., Gacser A., Mora-Montes H.M. (2016). Members of the *Candida parapsilosis* Complex and *Candida albicans* Are Differentially Recognized by Human Peripheral Blood Mononuclear Cells. Front. Microbiol..

[31] Walker L.A., Munro C.A., De Bruijn I., Lenardon M.D., McKinnon A., Gow N.A.R. (2008). Stimulation of Chitin Synthesis Rescues *Candida albicans* from Echinocandins. PLoS Pathog..

[32] Stappers M.H.T., Brown G.D., Prasad R. (2017). Host Immune Responses During Infections with *Candida albicans*. Candida albicans: Cellular and Molecular Biology.

[33] Díaz-Jiménez D.F., Pérez-García L.A., Martínez-Álvarez J.A., Mora-Montes H.M. (2012). Role of the Fungal Cell Wall in Pathogenesis and Antifungal Resistance. Curr. Fungal Infect. Rep..

[34] Lorenz A. (2022). Candida auris: Methods and Protocols.

[35] Bentz M.L., Sexton D.J., Welsh R.M., Litvintseva A.P. (2019). Phenotypic Switching in Newly Emerged Multidrug-Resistant Pathogen *Candida auris*. Med. Mycol..

[36] Bruno M., Kersten S., Bain J.M., Jaeger M., Rosati D., Kruppa M.D., Lowman D.W., Rice P.J., Graves B., Ma Z. (2020). Transcriptional and Functional Insights into the Host Immune Response against the Emerging Fungal Pathogen *Candida auris*. Nat. Microbiol..

[37] Vanden Bossche H., Kuhn P.J., Trinci A.P.J., Jung M.J., Goosey M.W., Copping L.G. (1990). Importance and Role of Sterols in Fungal Membranes. Biochemistry of Cell Walls and Membranes in Fungi.

[38] Shahi G., Kumar M., Kumari S., Rudramurthy S.M., Chakrabarti A., Gaur N.A., Singh A., Prasad R. (2020). A Detailed Lipidomic Study of Human Pathogenic Fungi *Candida auris*. FEMS Yeast Res..

[39] (2022). The Jamovi Project. Jamovi, Version 2.3. https://www.jamovi.org.

[40] R Core Team (2021). R: A Language and Environment for Statistical Computing, Version 4.1. https://cran.r-project.org.

[41] Fox J., Weisberg S. (2020). car: Companion to Applied Regression. [R Package]. https://cran.r-project.org/package=car.

