# Supplementary material for: Characterization and Differentiation of Candida auris on Dixon’s Agar Using Raman Spectroscopy
Source: Pathogens. 2024 Nov 8;13(11):978. doi: 10.3390/pathogens13110978 (PMC11597615; doi:10.3390/pathogens13110978)
Supplement: Supplementary file 1 [file pathogens-13-00978-s001.zip › pathogens-3302539-supplementary.pdf]

Characterization and Differentiation of *Candida auris* on Dixon's Agar Using Raman Spectroscopy

Supplementary information

This supplementary file contains the statistical analysis of the significant Raman band ratios for all *Candida* species included in the study. These ratios, which contribute to species differentiation in the relevant PCA results, provide a molecular basis for distinguishing between the different *Candida* species.

Descriptives

						Shapiro-Wilk	
	**Class**	**Mean**	**SE**	**Median**	**SD**	**W**	**p**
1452/1171 (1/cm)	*C. albicans*	4.34	0.432	4.40	1.06	0.964	0.852
	C. auris A	10.70	1.371	10.87	3.07	0.961	0.814

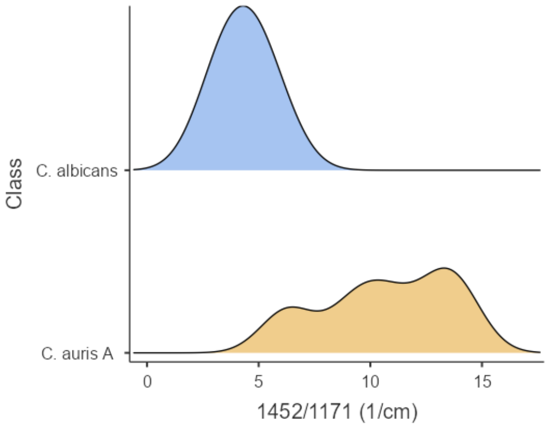

Density plot (Raman bands ratio)

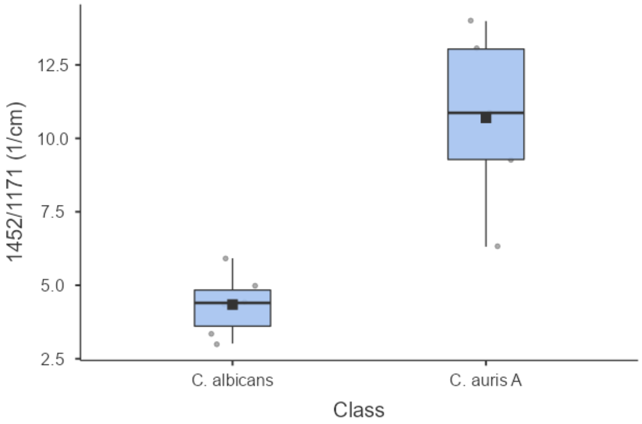

Box plot (Raman bands ratio)

Independent Samples T-Test

		Statistic	df	p	Mean difference	SE difference		Effect Size
1452/1171 (1/cm)	Welch's t	-4.42	4.80	0.008	-6.35	1.44	Cohen's d	-2.77

Note. H_a $\mu_{C. albicans} \neq \mu_{C. auris A}$

Normality Test (Shapiro-Wilk)

	W	p
1452/1171 (1/cm)	0.961	0.784

Note. A low p-value suggests a violation of the assumption of normality

Homogeneity of Variances Test (Levene's)

	F	df	df2	p
1452/1171 (1/cm)	4.65	1	9	0.059

Note. A low p-value suggests a violation of the assumption of equal variances

Q-Q plot

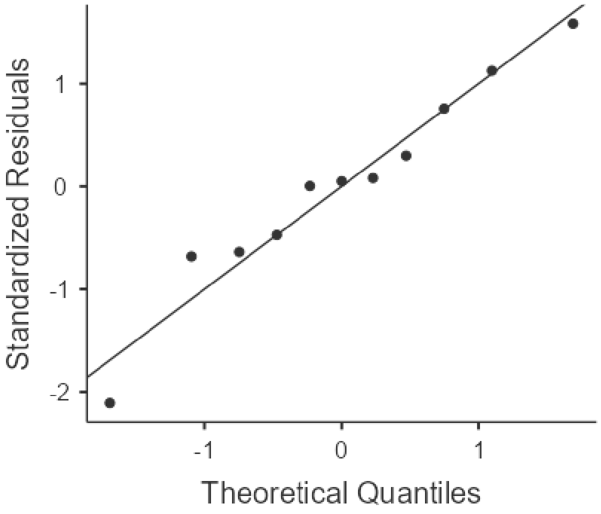

Descriptives

	Class	Mean	SE	Median	SD	Shapiro-Wilk	
						W	p
1452/1090 (1/cm)	*C. parapsilosis*	0.643	0.0394	0.665	0.1044	0.928	0.538
	C. auris B	0.536	0.0233	0.514	0.0903	0.903	0.106

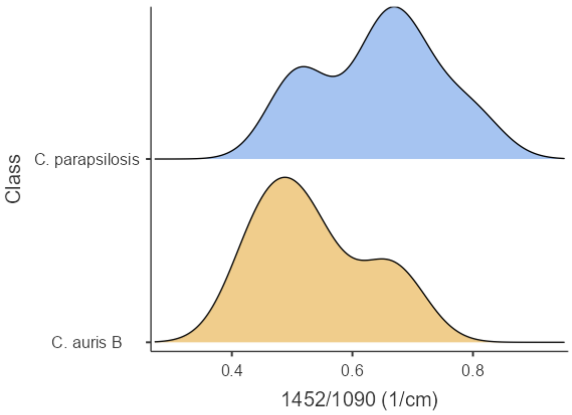

Density plot (Raman bands ratio)

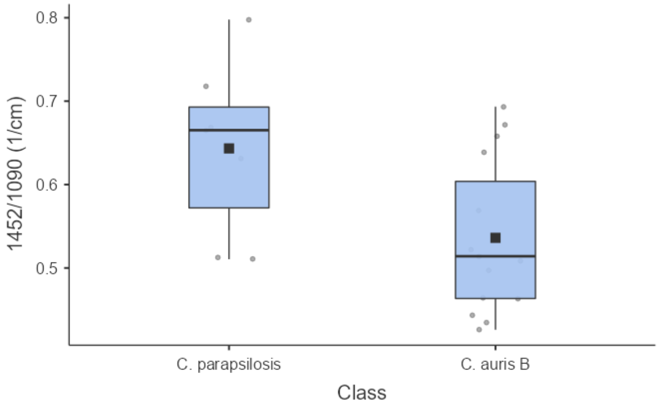

Box plot (Raman bands ratio)

Independent Samples T-Test

		Statistic	df	p	Mean difference	SE difference		Effect Size
1452/1090 (1/cm)	Welch's t	2.34	10.4	0.040	0.107	0.0458	Cohen's d	1.10

Note. H_a $\mu_{\text{C. parapsilosis}} \neq \mu_{\text{C. auris B}}$

Normality Test (Shapiro-Wilk)

	W	p
1452/1090 (1/cm)	0.942	0.217

Note. A low p-value suggests a violation of the assumption of normality

Homogeneity of Variances Test (Levene's)

	F	df	df2	p
1452/1090 (1/cm)	0.0444	1	20	0.835

Note. A low p-value suggests a violation of the assumption of equal variances

Q-Q plot

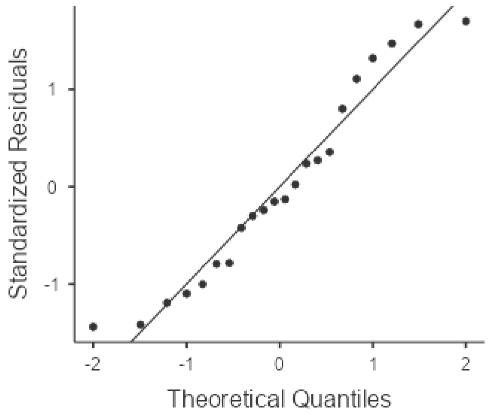

References
The jamovi project (2022). *jamovi*. (Version 2.3) [Computer Software]. Retrieved from https://www.jamovi.org.
R Core Team (2021). *R: A Language and environment for statistical computing*. (Version 4.1) [Computer software]. Retrieved from https://cran.r-project.org. (R packages retrieved from MRAN snapshot 2022-01-01).
Fox, J., & Weisberg, S. (2020). *car: Companion to Applied Regression*. [R package]. Retrieved from https://cran.r-project.org/package=car.
